# A new approach for national-scale Building Energy Models based on Energy Performance Certificates in European countries: The case of Spain

**DOI:** 10.1016/j.heliyon.2024.e25473

**Published:** 2024-02-01

**Authors:** Carlos Beltrán-Velamazán, Marta Monzón-Chavarrías, Belinda López-Mesa

**Affiliations:** Built4Life Lab, University of Zaragoza-I3A, 50018 Zaragoza, Spain

**Keywords:** Urban building energy Model (UBEM), Energy performance certificate (EPC), Cadastral data, GIS, Europe, National building stock

## Abstract

Urban Building Energy Models (UBEMs) are useful instruments to know the energy consumption of building stocks at urban and national levels. UBEMs can be classified into different types and subtypes. The current detailed physics-based bottom-up UBEMs at a national scale play a crucial role in assessing the energy efficiency of national building stocks and defining improvement strategies. These models heavily rely on archetypes and energy simulations, demanding significant computational resources. We propose here a new type of national-scale detailed physics-based UBEM based on Energy Performance Certificates (EPCs), and other open big data, which has the advantage that it can be automatically updated, in a short time, and with standard computer means. In this paper, we define the methodology to build this new type of national-scale EPC-based UBEM. We have checked that the model for the case of Spain can be automatically generated and updated in less than 6 h with a standard computer, and it generates results that match official data in more than 98 % for four indicators. The generated model contains information about 10,939,801 buildings in Spain, out of which 1,202,708 have EPCs. The model allows us to map and analyse the buildings in the country by integrating multiple variables of different nature, such as geographical (Autonomous Community, municipality, type of municipality), physical (area, number of floors, date of construction), use-related (main use of the building and use of each of its building units), and energy-related (climate zone, energy class, energy consumption, CO_2_ emissions). In this paper, we have proven that the model allows for the development of some indicators to measure the progress of decarbonisation trajectories whose development will become mandatory for European Member States soon.

## Highlights

• A new national-scale Urban Building Energy Model based on European open data
• Open data on Energy Performance Certificates cuts computer demand
• It generates insights for building renovation policy and planning
• Auto-updatable model, easily generated on a standard computer
• The model maps and analyses geographical, physical, usage, and energy data

## Abbreviations

2023 EPBD recast: 2023 amended version of the proposal for the Energy Performance of Buildings Directive recast
AC: Autonomous Communities
API: Application Programming Interface
BU: Building Unit
CESAR-P: Combined Energy Simulation And Retrofit in Python
CNIG: National Centre for Geographic Information
CO_2_ emissions: Carbon dioxide emissions
DDBB: Database
DTM: Digital Terrain Model
EPBD: Energy Performance of Buildings Directive
EPC: Energy Performance Certificate
EU: European Union
GHG: greenhouse gas
GIS: Geographic Information System
IGN: National Geographic Institute of Spain
INE: National Statistics Institute of Spain
INSPIRE: Infrastructure for Spatial Information in Europe
MEPS: Minimum energy performance standards
MS: Member States
NRPEC: Non-renewable primary energy consumption
UBEM: Urban Building Energy Model

## 1 Introduction

Within the European Union (EU), buildings play a significant role in combating climate change, factoring in 40 % of the Union's final energy consumption and 36 % of its energy-related greenhouse gas (GHG) emissions [1]. The goal in Europe is to ensure that all new constructions are zero-emission buildings, while also upgrading existing ones to meet this standard by the year 2050. Addressing the urgent need for decarbonisation in the EU's building inventory requires a substantial increase in energy renovations, especially considering that almost 75 % of current buildings fall short of efficiency standards, and 85–95 % are estimated to remain in use by 2050 [1]. Despite this objective has been outlined in the Energy Performance of Buildings Directive (EPBD), the annual energy renovation rate has persistently lingered at around 1 %. If this slow pace persists, the decarbonisation process for the building sector could span centuries. Recognizing this situation, the 2023 amended version of the proposal for the recast of the Energy Performance of Buildings Directive (referred to as 2023 EPBD recast in this context) sets a crucial objective of stimulating and supporting building renovations, with the aim of at least tripling the current renovation rate [1]. At the heart of this initiative are the Minimum Energy Performance Standards (MEPS), serving as crucial regulatory tools to drive extensive renovations of existing buildings. Their implementation is expected to facilitate the diminishment of the least energy-efficient buildings while encouraging continuous improvement of the whole national building stock [1].

The 2023 recast of the EPBD stipulates that Member States (MSs) must define a linear trajectory to progressively attain higher energy performance classes, ensuring that.

• Publicly owned buildings and building units (BU) must attain a minimum efficiency class of E by January 2027 and progress to class D by January 2030.
• Non-residential buildings and BU achieve at least performance classes E and D as of January 2027 and 2030, respectively.
• Buildings and BU of residential buildings achieve at least class E and class D as of January 2030 and 2033, respectively.

The European Energy Performance Certificates (EPCs) classify buildings between A (Very efficient) and G (Very inefficient), therefore, E, F, and G correspond to the worst-performing segments of each country. MSs must have a diagnosis of the energy performance of their stock of buildings and establish a renovation trajectory to achieve the MEPS set by Europe.

The existing literature identifies methodologies to recognise priority buildings to be renovated among certain types of buildings and urban areas, e.g. Refs. [[2], [3], [4]]. These studies are useful for prioritizing buildings to renovate, however they focus only on certain building typologies and small areas. Therefore, they are not useful for a national scale prioritization.

Urban Building Energy Models (UBEMs) have been identified as useful tools to know the energy consumption of urban areas or cities for sustainable urban development and improvement [5]. UBEMs are energy models at district, city or national scale [6], which can be categorized into two main types depending on how the model is generated [7,8].

• Top-down models use mostly macroeconomic variables, such as historical time series to determine energy predictions, mainly based on interrelationships between the energy sector and the broader economy. Detailed building information is not necessary.
• Bottom-up models depict energy consumption by utilizing detailed end-use information. They can be divided into two subtypes:
  o Statistical bottom-up models: they rely on data that characterizes the energy usage of individual buildings.
  o Physics-based bottom-up models: they use the physical characteristics of buildings to simulate their energy consumption. They require energy modelling of buildings. They can also be divided into [9]:
    ▪ Simplified physics-based bottom-up models: they use simplified engineering models for energy estimations. In this method, determining the indoor and outdoor heat exchange entails considering elements such as enclosure thermal efficiency, external air conditions, and internal control variables [9].
    ▪ Detailed physics-based bottom-up models: they use detailed engineering models for energy estimations with the support of simulation tools such as EnergyPlus [9].

In large-scale analysis, physics-based bottom-up methodologies are critical in identifying potential efficiency improvements for urban development and the construction sector [6] since they can be used to analyse and evaluate projected futures and technological innovations [10]. This is why they are an appropriate approach to establish a renovation trajectory to achieve the MEPS set by Europe. Simplified engineering models lack the precision of detailed engineering models. However, one of the challenges that detailed physics-based bottom-up UBEMs currently have is to reduce computer resources and time-consumption necessary to generate these models [11]. These detailed physics-based bottom-up approaches lies on modelling individual buildings or building archetypes, i.e. representations of a group of individual buildings through the use of a typical building. The use of archetypes is a strategy to simplify the modelling of entire districts, cities or countries, via detailed building simulation models for the archetypes [10]. It has been found that the use of this representative building approach reduces modelling work and computation and yields comparable outcomes in terms of mean aggregated energy savings, although the actual variation is underestimated due to the absence of diversity in building characteristics [12].

Whereas UBEMS at district and city scales are representative in the literature, country-scale UBEMS are few in number and they are mainly based on representative-building detailed physics-based bottom-up approaches. Despite the simplification by the use of archetypes, they are computational resource demanding because they require the consideration of the buildings of a whole country. Berres et al. [13] propose a methodology to simulate 125 millions of buildings in the US based on building standards and building types, using a supercomputer, called AutoBEM. The Urban Energy Systems Lab in the Swiss Federal Laboratories for Material Science and Technology (EMPA) has developed a software called Combined Energy Simulation And Retrofit in Python (CESAR-P) that studies the energy performance of buildings in Switzerland by means of building archetypes and Energy Plus simulations [14]. The CESAR-P model requires a supercomputer to perform the calculation and generates a very detailed information in a building-by-building model of Switzerland. These two national-scale representative-building detailed physics-based bottom-up UBEMs have a high potential to diagnose national building stocks, however they require an intensive use of computational resource. The AutoBEM model is automatically updated as new data is available, requiring for this the use of a supercomputer to update the model. The CESAR-P model uses geospatial data, allowing to generate multiple analyses that are not possible on tabular data [15], however it also requires a supercomputer for the simulations. Additionally, since these national models require the generation of archetypes, only the most common cases of every cluster are considered and for the rest of cases the same behaviour is assumed.

**Fig. 1:**
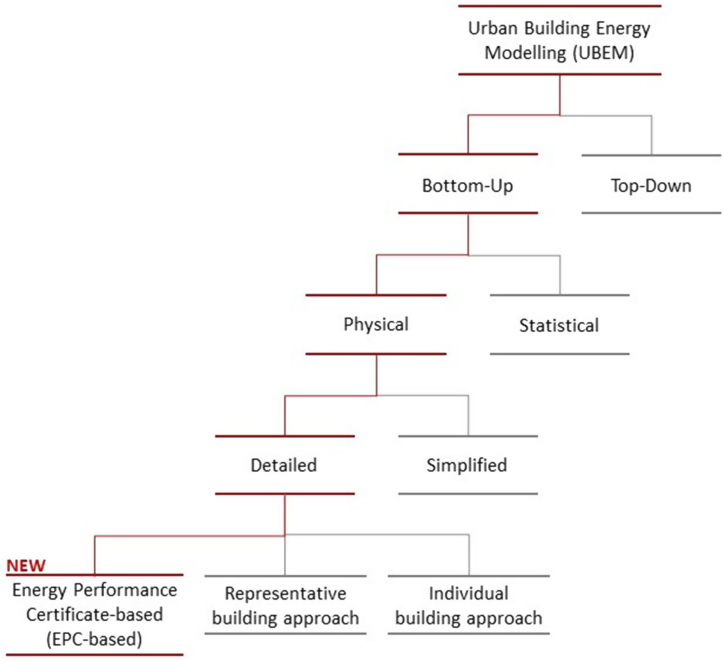
Subgroup of existing methods for UBEMs, path chosen in this paper, and new approach proposed for national-scale UBEMs.

In this paper we propose a new approach for national-scale detailed physics-based bottom-up UBEMs with reduced computational resource demand, based on the use of Energy Performance Certificates (EPC) and other open data. The EPC serves as a rating system designed to condense information about the energy efficiency of buildings, with the goal of offering consumers insights into properties they intend to buy or lease in European countries. The scheme is regulated by Directive 2010/31/EU [16]. Member States apply an energy simulation methodology to assess the energy performance of buildings, adhering to the common general framework outlined in Annex I of the mentioned Directive. In the case of Spain, the methodology to simulate and certify the energy efficiency of buildings is defined in Royal Decree 390/2021 [17], Royal Decree 450/2022 [18], and a Procedure defined by the Spanish Government [19], and it is based on UNE-EN ISO 52000-1 [20]. The main novelty of our model with respect to the existing national-scale detailed physics-based bottom-up UBEMs is that it is not based on the simulation of archetypes nor on the simulation of buildings one-by-one, but on the available EPC results for which detailed simulations of buildings or BUs have already been performed. EPCs can be useful to solve the problem of intensive use of computational resource because they are created in a building-by-building or BU-by-BU case basis and already incorporate detailed energy information developed by different technicians following a well-defined methodology and official software. This allows us to do a model which contains the detailed and specific characteristics of a high number of buildings, with less intensive computational resource demand. It also overcomes the disadvantage of underestimation of the actual spread of energy assessments based on representative buildings since the number of certified buildings is high and increasing. We have named our model approach as Energy Performance Certificate-based Urban Building Energy Model (EPC-based UBEM). The use of Energy Performance Certificates (EPCs) has been identified as the appropriate tool by the European Commission to diagnose buildings energy efficiency, and they continue promoting its use through the directives. The EPCs represent a free resource available to MS that offers detailed energy information about the building stock with high potential in combination with other sources of information. Fig. 1 shows a summary of some of the existing approaches for UBEMs in the literature. We have highlighted red the path chosen in this paper. The main novelty of this paper is that we propose a new approach for national-scale detailed physics-based bottom-up UBEMs, which is less computational resource demanding, and will be useful to define renovation trajectories of building stocks in European countries.

Other characteristics of the model we propose here are that: a) we make use of Geographic Information System (GIS) open data, and tabular open data which we georeference; and b) our model is designed to be automatically updated, being this specially important when the information is very disjointed as automation provides the advantage of allowing its permanent updating. Table 1 summarises the novelties of our model in relation to other existing national-scale UBEMs.

**Table 1:** Comparison of characteristics of national-scale UBEMs.

Model name	Model generation approach	Data type generated	Automatically updated	Computer requirements
AutoBEM	Representative building approach (Archetype + simulation)	Tabular data	Yes	Supercomputer
CESAR-P model	Representative building approach (Archetype + simulation)	GIS + tabular data	No	Supercomputer
EPC-based UBEM (present study)	EPC-based	GIS + tabular data	Yes	Standard computer

The main objective of this paper is to define a new type of national-scale UBEM based on EPCs. Additionally, as suggested by other authors [21], we use other big open data to enrich the energy model. The model construction is done in an automated way, using open data from sources established at a European scale and other national open data, to build a single GIS database (DDBB) that allows for a diagnosis of the national building stock that is useful to establish national renovation trajectories in European countries. This objective will be developed for the case of Spain in this paper. For the energy diagnosis of national building stocks, the model should allow to.

• Know the general characteristics of the buildings/buildings units (BU) depending on whether they are non-residential or residential buildings: year of construction, number of floors, use, square meters, location, climate zone, etc.
• Identify the energy characteristics of buildings/buildings units depending on whether they are non-residential or residential buildings: energy performance classes, non-renewable primary energy consumption (NRPEC), CO_2_ emissions, certified square meters.

All this information will be crucial in defining the trajectory for the gradual improvement of the energy performance of national building stocks in Europe, as stipulated by the 2023 recast of the EPBD [1].

## 2 Materials and methods

### 2.1 Materials

The availability of open data on buildings and the possibility of sharing them have become a key in order to achieve decarbonisation objectives at a European level [22]. In European countries, three of the most common public information repositories on existing buildings are EPCs, cadastre and census. EPCs and cadastre provide information which can be useful to define the linear trajectory for the gradual transformation of the building stock into buildings with higher energy performance classes. The census provides complementary data, such as the municipality size, or socioeconomic data that allow to enrich the models. Next, we will further study the two European databases that can be useful when defining renovation trajectories.

#### 2.1.1 Energy performance certificates (EPCs)

Energy Performance Certificates (EPCs) are an important instrument to enhance the energy efficiency of buildings in Europe, serving as a key instrument within the framework of the Energy Performance of Buildings Directive [16].

The EPC is a tool with a high potential since its information must be publicly accessible in compliance of the Energy Building Performance Directive (EPBD). The EPDB of 2010 and 2018 already regulated that the Energy Performance Certificate databases (EPC DDBBs) should be available for research on request. However, the 2023 proposal goes further and specifies that the EPC DDBBs must be publicly available, machine-readable, accessible via a digital interface and interoperable with administrative DDBBs such as cadastre or digital building logbook. This will greatly enhance the potential of EPCs for research across Europe. So far, EPCs have already been used in many research studies. According to Pasichnyi et al. [23] 79 papers used EPCs until 2018. They have been used for regional, e.g. Refs. [[24], [25], [26]], and country-scale studies, e.g. Refs. [[27], [28], [29]]. All these studies are of great interest for our research goal, however, the results from these studies do not differentiate by climate zone or region, nor are they designed to identify priority areas to renovate, and some of them are just applied to some types of buildings.

Currently, the DDBBs availability varies from one country to another. Spain currently offers a large part of its EPC open. In countries like Spain or Italy, where the EPCs are managed by the different Autonomous Communities (ACs) or regions [30], the EPCs information is made available in different format in each AC. For this reason, we consider Spain an interesting case study.

The country of Spain is divided into 17 ACs or regions, and 2 cities with autonomy statute. The ACs are subdivided into provinces. Some studies using EPCs have already been conducted in Spain at region scale, e.g. Refs. [[31], [32], [33]]. These studies are also of great interest for us, however, they focus only in one region of Spain and represent insights outdated now. Recently, the Spanish Government published URBAN3R [34] an open data platform to promote urban regeneration in Spain. It uses cadastral data and GIS, to estimate the heating demand of buildings using clusters from 750 case studies. At this moment, the platform only includes the main cities.

Therefore, we can conclude that there is an increasing use of EPCs in the literature and that our national-scale EPC-based UBEM model is novel since there is no other model using EPCs that simultaneously meet the following criteria: it is at a country-scale and the methodology to build it can be applied to different European countries, it includes all types of buildings, it is combined with other sources to provide enriched data useful for the energy diagnosis and design of renovation trajectories, and it can be automatically updated.

#### 2.1.2 Cadastral data

The cadastre is a detailed administrative register containing information about the boundaries, dimensions, and characteristics of the real estate across a country. For several years in the EU there has been an effort to share information in a transparent and accessible way for citizens and to do it in a common and homogeneous way [35]. The Infrastructure for Spatial Information in Europe (INSPIRE), regulated through its Directive [35], serves as a useful instrument in addressing policies and actions with a direct or indirect influence on both cities and the environment. The INSPIRE cadastres are georeferenced dataset maintained and produced by the registers of MS to comply with the INSPIRE Directive, so that they are compatible and useable in a community and cross-border context.

Alphanumeric and INSPIRE cadastral data have been used by other authors to obtain energy building information, e.g., Refs. [21,36,37]. In conclusion, alphanumeric and INSPIRE cadastral data enhance the energy models through the incorporation of detailed information about the characteristics of the buildings.

### 2.2 Research methodology

**Fig. 2:**
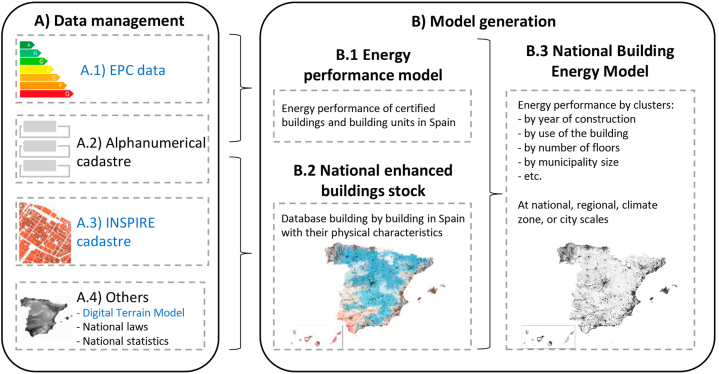
General methodology flowchart to build a national-scale EPC-based UBEM in European countries. Data available at European level in blue. Complementary national data in black. (EPC: Energy Performance Certificate; INSPIRE: Infrastructure for Spatial Information in Europe). (For interpretation of the references to colour in this figure legend, the reader is referred to the Web version of this article.)

We first built the national-scale EPC-based UBEM for the case of Spain following the general methodological scheme of Fig. 2. This scheme is applicable to all European countries and is based on European open data (the INSPIRE cadastre, the open data of EPCs and the Digital Terrain Model (DTM)), in addition to other complementary open data information to enrich the analysis (Fig. 2).

The model construction was automated and allows automatic updating. The methodology was based on two main blocks: Block A) Data management; and Block B) Model generation. Block A included downloading, data transformation and filtering of errors and outlier values. Block B included data pooling, the creation of the models, and generating data grouped in clusters to analyse them.

To carry out the process of transformation, combination and analysis of the data, the software used is specified next.

• Code editor and language: Visual Studio Code and Python (version 3.11.1).
• Data analysis tools in Python: Pandas v.1.5.3, Numpy v.1.24.1, Polars v.0.16.13, Xlrd v.2.0.1, Elementpath v.4.0.1, Simpledbf v.0.2.6.
• Geographic analysis tools: QGIS Desktop v.3.28.3, Geopandas v.0.12.2. Utilizing a GeoPandas-based approach has been proven advantageous when handling extensive geographical datasets [38].

After the construction of the model, we tested it and studied its usefulness. The model was tested by assessing the time consumption to build or update the model, and by analysing the robustness of the model by means of verifying the ability to obtain correct results for main indicators.

To test the time consumption to generate the national-scale EPC-based UBEM, the model for the whole country of Spain was generated using a standard computer (Processor 11th Gen Intel(R) Core(TM) i5-11400F @ 2.60 GHz 2.59 GHz; 16 GB RAM; Windows 11, 64-bit operating system). The country used as case study, Spain, has an extension of 505,990 km^2^ [39], 47,615,034 inhabitants [40], and 10,939,801 buildings which occupy a total area of 3835.5 km^2^.

To analyse the robustness of the model to obtain correct results, four indicators were used. The model results for those indicators were compared to official data. These indicators are.

• Indicator 1, number of buildings, as compared to the statistics of the national cadastre of Spain.
• Indicator 2, number of dwellings, as compared to the statistics of the national cadastre of Spain.
• Indicator 3, number of real estates, as compared to the statistics of the national cadastre of Spain.
• Indicator 4, percentage of buildings with different energy classes, in comparison to the officially reported national data on the Energy Certification status of buildings [41].

The usefulness of the model was finally studied by means of the generation of new data on the building stock of Spain. For this, we selected some indicators that the coming European Directives will soon establish as mandatory indicators to be developed by the Member States in order to measure the progress of the renovation trajectories [1].

• Average non-renewable primary energy use in kWh/m^2^y.
• Annual greenhouse gas emission per building type.
• Number of EPCs per energy class and number of EPCs per building type.
• Number of buildings and square meters per energy performance class.

#### 2.2.1 Methodology to build the national-scale EPC-based UBEM for the case study of Spain

Spain is an interesting case study for the application of the new approach for national-scale detailed physics-based bottom-up approach of UBEMs, as this country has many competences delegated to the ACs. This means that many of the data cannot be obtained from a unified national source but must be obtained from each of the ACs. For the case of Spain, the data used, and the sources of information are shown in Table 2.

**Table 2:** Data used and its source at the case of Spain. MDT_200: Digital Terrain Model, AC: Autonomous Communities.

Data	Source	Type of source	Update frequency
EPC databases	Different for each AC in Spain (see Appendix A)	Tabular data	Vary by AC
INSPIRE cadastral data	INSPIRE Cadastral Cartography Services [42]	GIS map	Every 6 months
Alphanumerical cadastral data	Electronic Office of the Cadastre [43]	Tabular data	Updated at download time
MDT_200	Directorate-General of the National Geographic Institute (IGN) of Spain [44]	GIS map	Every 5 years approx.
Climate zones	CTE DB HE “Ahorro de energía" [45]	Spain regulation on Energy Savings	Current legislation
Efficiency classes according to energy consumption and emissions	Document: Qualification of the energy efficiency of buildings, Version 1.1/November 2015, Ministry of Industry, Energy and Tourism [46]	National regulation	Current legislation
Population census by municipality	National Statistics Institute (INE) [47]	National statistics	Every year

To validate an EPC in Spain, its information has to be registered in the department of the corresponding AC, which can be found in Table A.1in Appendix A. EPCs are mandatory in Spain for existing buildings that are rented or sold since June 2013 [48]. The certification is valid for ten years, although the owner may voluntarily update the EPC when a renovation changes the energy class obtained. When registering an EPC, technicians have to upload a xml file generated by the certification software, which is the same for the whole of Spain. However, there is no regulation on what information the ACs must publish. For this reason, the information published and the way of presenting it are uneven, giving rise to multiple incompatibilities and information gaps.

The Spanish cadastre makes open data available to the citizen, such as alphanumeric information, vector information, and vector information through the INSPIRE cadastre. Vector information is georeferenced while the alphanumeric information is not. The regions of Navarra and Basque Country are neither included in the INSPIRE cadastre nor in the Spanish cadastre, so they could not get included in this study.

**Fig. 3:**
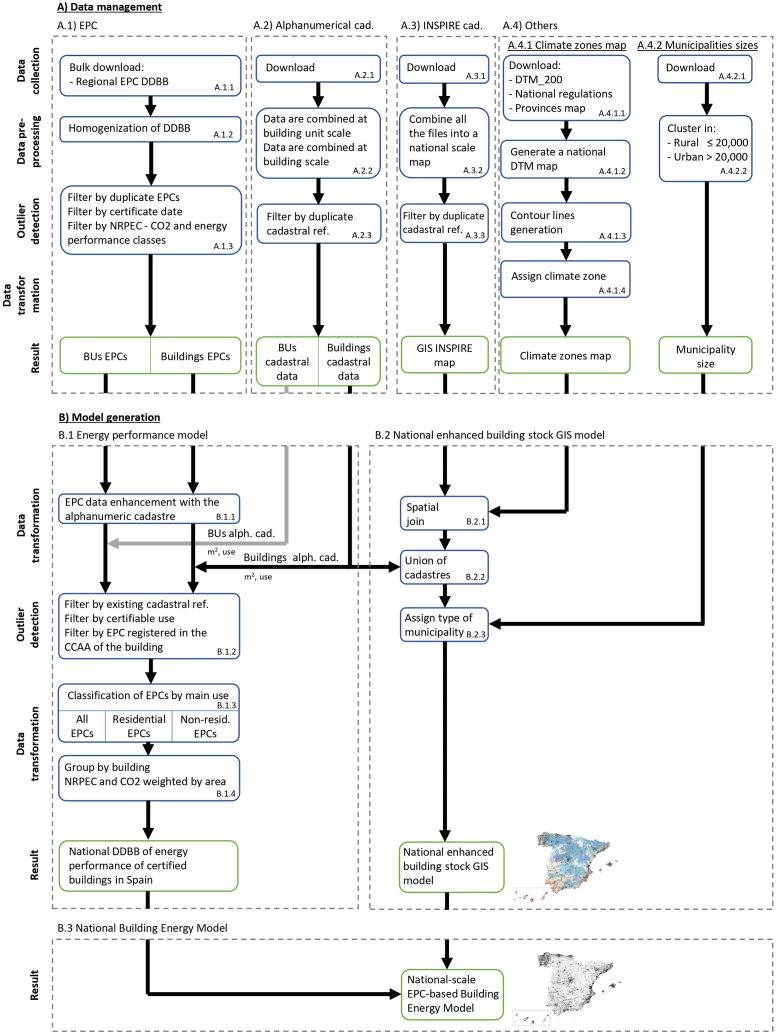
Flowchart of the specific methodology to build the national-scale EPC-based UBEM for the case of Spain. Processes in blue. Results in green. (Alph.: alphanumerical; BUs: Building Units; Cad.: cadastre; DDBB: Databases; DTM: Digital Terrain Model; EPC: Energy Performance Certificate; GIS: Geographic Information System; INSPIRE: Infrastructure for Spatial Information in Europe; NRPEC: Non-renewable primary energy consumption; Non-resid.: non-residential). (For interpretation of the references to colour in this figure legend, the reader is referred to the Web version of this article.)

The methodology to build Spain's EPC-based UBEM followed the detailed workflow indicated in Fig. 3. It is composed of two main steps.

• **Step A – Data management.** It consists of data collection, data pre-processing, outlier detection, and data transformation of four different DDBBs. These DDBBs are:
  o A1. EPC
  o A2. Alphanumeric cadastre
  o A3. INSPIRE cadastre
  o A4. Others.
• **Step B – Model generation.** Taking advantage of the synergies of combination of the data from the different sources of information, three models were generated:
  o B1. Energy performance model. It is a database of the energy performance of all the certified buildings and BU in Spain. This model contains the NRPEC and CO_2_ emissions of each building and BU holding an EPC, its energy class, and certified surface.
  o B2. National enhanced building stock GIS model. It is a GIS model of all the buildings in Spain with their general characteristics. This model is georeferenced and contains detailed information about its use, built-up area by use, year of construction, climate zone, etc.
  o B3. National-scale Building Energy Model based on Energy Performance Certificates (national-scale EPC-based UBEM). It is the combination of both models. The result is a GIS map and a database with information on all certified buildings and BUs, having both types of information, their energy performance and their general characteristics. The importance of this model is that it allows determining the representativeness of certified buildings at the national level and grouping them in clusters and analysing them.

Further details about the data provided by the DDBBs we used to generate the models (EPC, alphanumeric cadastre, INSPIRE cadastre, and others) can be found in Table A.2 in Appendix B. Additionally, in Table A.3 in Appendix C, we show the data that our three models provide. Steps A and B are subdivided in sub-steps which are further developed next.

**Table 3:** Time consumption to automatically generate the models (EPC: Energy Performance Certificate).

Step	Output data	Time consumption		
A.1 EPC	3,920,354 EPCs	2h	30 min	
A.2 Alphanumerical cadastre	11,120,066 buildings	1h	3 min	53 s
A.3 INSPIRE cadastre	12,340,420 buildings	2h		
A.4.1 Climate zones map	505,990 km^2^	Non-automated		
A.4.2 Municipality size	8131 municipalities			4 s
B.1 Energy performance model	2,966,479 EPCs		4 min	40 s
B.2 National enhanced building stock GIS model	10,939,801 buildings		9 min	30 s
B.3 National-scale Urban Building Energy Model based EPCs	10,939,801 buildings out of which 1,202,708 have EPC		1 min	20 s
Total		5h	49 min	27 s

##### 2.2.1.1 Step A1 - EPC data management

The EPC data are published in open access by 13 ACs, which in practice means 13 different ways of providing the information, as was already identified by Terés-Zubiaga et al. [31]. Therefore, the aim of this step is to transform and analyse the EPC data to obtain a common and homogeneous DDBB at national level that allows us to analyse the energy performance data of the certified buildings in Spain. Due to the disparity of data provided by each Spanish AC, it was decided to take the minimum essential data from the EPCs. This step was composed of the following sub-steps.

• **Step A.1.1 - EPC bulk download.** The information from the different ACs was downloaded. In Table A.4 in Appendix D, the different EPC information in open data provided by each AC is shown.

**Table 4:** Time consumption to read the models files using Pandas 1.5.3 and Geopandas v.12.2 and the size of the files.

Step	Time consumption: csv format	Size of the models: csv	Time consumption: parquet format	Size of the models: parquet
B.1 Energy performance model	8.8 s	719 MB	1.4 s	75 MB
Step	Time consumption: shp format	Size of the models: shp	Time consumption: geoparquet format	Size of the models: geoparquet
B.2 National Enhanced building stock GIS model	13 h	28.5 GB	4.5 min	2.8 GB
B.3 National Building Energy Model	19.4 min	9.5 GB	6.8 s	487 MB
• **Step A.1.2 – EPC data pre-processing.** The information published by the ACs differs not only in the data, but also in the format. Because of this, the data was pre-processed. Table A.5 in Appendix E shows the different file formats published by each AC. The information provided by each AC was standardised by organising it according to a homogeneous set of data, so that they can be analysed as a whole. This study uses the csv and parquet format with UTF-8 encoding, having to parse characters not supported in other formats, and transform the languages based on hierarchical levels to data in csv format in order to be able to study them as a whole.

**Table 5:** Comparison of number of buildings, dwellings and real estates in the national-scale EPC-based and in official statistics [55].

Indicator	National-scale EPC-based UBEM	Official data	Relative error
1. No. of buildings	10,934,241	11,130,090	1.8 %
2. No. of dwellings	23,690,136	23,238,488	1.9 %
3. No. of real estates	36,581,505	36,612,402	0.1 %
• **Step A.1.3 – Outlier detection in EPCs.** Some EPCs have incorrect or incoherent information as also detected in other studies [49]. For this reason, outlier detection was performed following the criteria in Table A.6 in Appendix F. With this filtering, 12.7 % EPCs were eliminated and 3,423,239 remained.

**Table 6:** Comparison of percentage of buildings with different energy classes between our model and official data [41] (NRPEC: non-renewable primary energy consumption).

Energy class	NRPEC			CO_2_ emissions		
	National-scale EPC-based UBEM	Official data	Error	National-scale EPC-based UBEM	Official data	Error
A	0.5 %	0 %	0.5 %	0.7 %	0 %	0.7 %
B	1.1 %	1 %	0.1 %	1.3 %	1 %	0.3 %
C	3.6 %	4 %	0.4 %	4.8 %	5 %	0.2 %
D	10.0 %	11 %	1.0 %	14.1 %	14 %	0.1 %
E	53.6 %	52 %	1.6 %	56.3 %	55 %	1.3 %
F	11.2 %	11 %	0.2 %	10.3 %	11 %	0.7 %
G	20.0 %	21 %	1.0 %	12.5 %	14 %	1.5 %

As a result of step A1, a DDBB with the EPCs of the national territory in the same standardised format was obtained, both at Building and BU scales.

##### 2.2.1.2 Step A2 - national alphanumeric cadastre data management

The information provided by the alphanumeric cadastre has a high level of detail, even if not georeferenced [50]. It provides information at various scales [43,51].

• Parcel, i.e. the cadastral plot;
• Construction Unit: it represents a building or a set of constructions within a building;
• Real estate: each of the real estates within a cadastral parcel.
• Construction: each of the existing parts in a real estate.
• Common elements.
• Croplands.

Among these six cadastral data categories, two of them are the main ones because they have assigned a cadastral reference: parcels and real estates. Parcels are identified with 14-digit cadastral references, and real estates with 20-digit cadastral references. Construction units do not have a specific cadastral reference, but the cadastre shows to what parcel they belong to through its cadastral reference. Constructions, common elements, and croplands do not have a specific cadastral reference either, however the cadastre displays to what real estate they belong to. The cadastral reference is very important in this study, since it is the reference that allows the connection between the alphanumeric cadastral data, the INSPIRE cadastral data, and the EPC data. Among these six cadastral data, we used the first four ones because they are the ones which provide data about buildings. Further details about the information useful for this study obtained from each of these four categories in the alphanumeric cadastre of Spain can be found in Table A.7 in Appendix G.

Step A2 was composed of the following sub-steps.

**Fig. 4:**
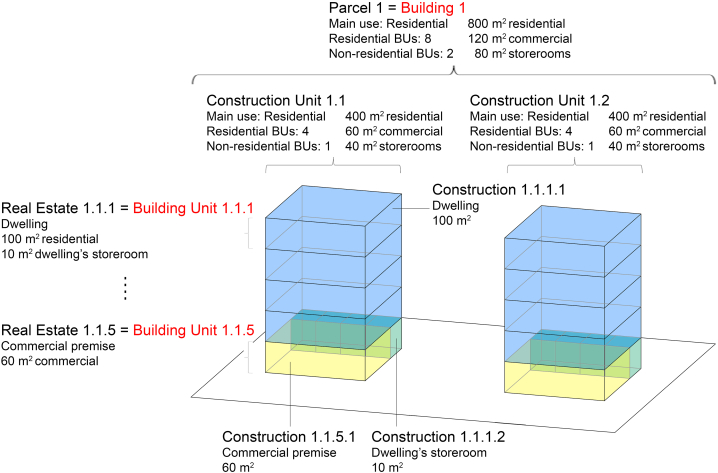
Example of a parcel to illustrate the relationship between the alphanumeric cadastral categories (in black) and the two EPC-based UBEM data scales (in red). (For interpretation of the references to colour in this figure legend, the reader is referred to the Web version of this article.)
• **Step A.2.3: Data cleaning.** In theory, cadastral references are unique and unequivocally identify a real estate or a cadastral parcel. However, some cadastral references have been found to identify more than one parcel or real estate. Since this is an error, they were eliminated from this study. A total of 7423 cadastral references were eliminated for this reason.

• **Step A.2.1 – Alphanumeric cadastre data bulk download.** The Spanish cadastre does not have an Application Programming Interface (API) that would allow downloading the alphanumeric data directly, and the bulk download is limited to xml queries of up to 250 Kbytes, equivalent to consulting approximately 5,500 cadastral references. Since March 16, 2023, it is possible to download all the information at the national level from the alphanumeric cadastre by provinces. For these reasons, the alphanumeric cadastral data bulk download was performed downloading the information package manually for each of the 52 Spanish provinces. However, the extraction and data management were automated.
• **Step A.2.2: Grouping of data at building and building unit scales.** Since the MEPS in the 2023 EPBD recast are applied to buildings or BUs, we grouped all the information into these two data scales. The BU is not specifically defined in the EPBD. In this paper, we understand that a BU is a property of a part of a building, either residential or non-residential. For example, in a multi-apartment building, a dwelling and a commercial office on the ground floor are different BUs. The categories that we use from the cadastre to enrich these two data scales can be found in Table A.8 in Appendix H. Fig. 4 shows the relationship between the alphanumeric cadastral categories and our model data scales. As can be seen in Fig. 4, when in a parcel there are two buildings we cannot differentiate between them. This is due to the fact that construction units in the alphanumeric cadastre do not have their own cadastral reference, but the parcel's one. For this reason, in our model a building is considered a building or set of buildings included in the same cadastral parcel. Finally, it must me noted, that since the MEPS in the 2023 EPBD recast differentiates between residential and non-residential buildings, we must assign a main use to each building in our model. The information about the main use of the building and BU is added considering the main use as the use that occupies the largest area (see example in Fig. 4).

As a result of step A2, we obtained two very detailed alphanumeric cadastre datasets, one at the building scale and another one at the BU scale.

##### **2.2.1.3** Step A3 – INSPIRE cadastre data management

The data management of INSPIRE cadastre was composed of the following steps.

• **Step A.3.1 - Bulk download from INSPIRE cadastre.** The main advantage offered by vector information from the INSPIRE cadastre compared to the vector information of the alphanumeric cadastre is that it has ATOM service, which allows the massive download of all the data in an easy and automated way. As the information is downloaded through a link per municipality, and Spain has more than 8,000 municipalities, the automation through ATOM allows the download of data of the entire country in a short time. To download all the information we generated a script, which allows to: 1) automatically download the information from the ATOM service; 2) extract the downloaded files in.zip format; 3) select the files we want to use, which, in this case, are the files named building.gml; and 4) join them together creating a shapefile containing all the information of the buildings at national scale. This allows to perform national scale analysis with GIS tools.
• **Step A.3.2 – Combination of files on a national scale.** Once the bulk download from INSPIRE cadastre was realised, the cadastral files were combined into a national scale map in which every building is georeferenced in a single file.
• **Step A.3.3 – Filtering of cadastral references.** As with the alphanumeric cadastre, there are cadastral references that identify two or more buildings. As this is a cadastral error, they are eliminated from this study. The total number of eliminated buildings was 9,936 for this reason.

As a result of step A3, we obtained a GIS map with all the buildings of Spain georeferenced.

##### 2.2.1.4 Step A4 - Additional data management

The additional data management included.

**Fig. 5:**
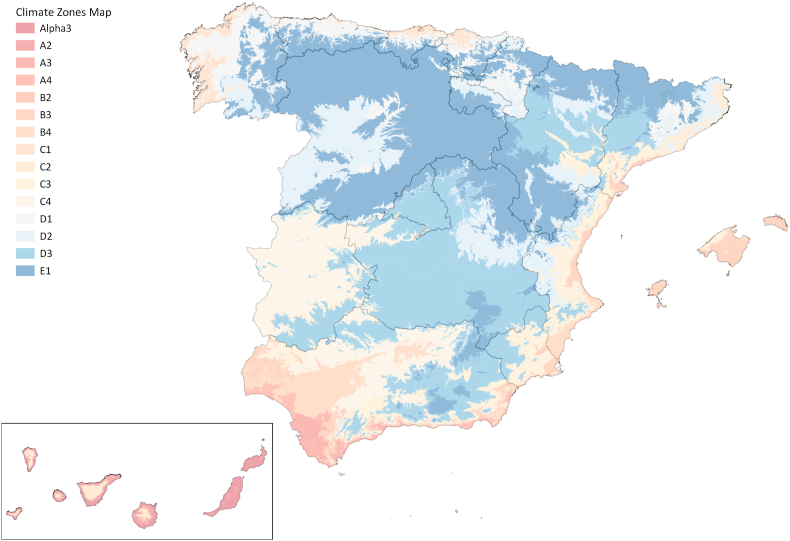
Climate zones in Spain in GIS.
• **Step A.4.2 – Municipality size.** According to the proposal for the EPBD recast, MEPS should design a pathway, supported by financial mechanisms, for the progressive improvement of the energy performance classes of buildings, with a specific focus on rural and isolated areas. For this reason, it is convenient to include in our model information about the population size of the municipalities, which allows us to distinguish between rural and urban areas. The National Statistics Institute (INE) of Spain provides information about the distribution of the inhabitants in the different municipalities of Spain. The INE assigns a code to each municipality. This code is also included in the parcel information in the alphanumeric cadastre. This allows us to assign to each building, municipality information from the INE, such as the size of the municipality in which it is situated. Then, we classified municipalities in rural (up to 20,000 inhabitants) and urban (from 20,001 inhabitants on).

• **Step A.4.1 – Generation of the climate zones maps.** As in Spain, national public administrations do not provide climate zones in GIS, one of the steps to build the national-scale EPC-based UBEM was to create a GIS map showing the climate zone of each building in the country. In Spain, the energy performance of buildings is regulated by the so-called Basic Document on Energy Saving (DB-HE) of the Technical Building Code (TBC or CTE for the initials of its name in Spanish). This regulation defines 17 climate zones in Spain, out of which 12 are situated in the peninsula and Balearic Islands and 5 in the Canary Islands. In Spain the CTE-DB-HE [52] defines the climate zones of each plot in Spain based on two criteria: the province in which it is located and the height above sea level (every 50 m). The climate zones are composed by a letter to classify the winter conditions (ranging from A to E, and α), and a number to categorise the summer conditions (from 1 to 4) [53]. With the purpose of creating a GIS map of climate zones we proceeded as follows:
  o A bulk download of the Digital Terrain Model (DTM) was realised. Specifically, the files of a model named ‘DTM first coverage with a 200-m mesh pitch (MDT_200)’ were downloaded from the Directorate-General of the National Geographic Institute (IGN) of Spain [44]. These files use the coordinate systems ETRS89 in the Peninsula, Balearic Islands, Ceuta and Melilla, and REGCAN95 in the Canary Islands. Both of them are compatible with WGS84. To generate the model, the information was used in UTM zone 30 which covers the entire peninsula and zone 28 for the Canary Islands. These files in asc format (Arc/Info ASCII Grid) were treated with QGIS software to combine them and create a complete map with all the DTMs of Spain (step A.4.1.2). This new model allows us to generate the contour lines of the terrain of the whole country every 50 m to determine the climate zone of each area (step 4.1.3). This file provides the altitude of each plot of Spain.
  o Then, the information of the National Centre of Geographic Information (CNIG) of the provinces of Spain was downloaded (step A.4.1.1). The provinces of Spain are obtained from the file called "Municipal, provincial and autonomic boundaries" available in the CNIG Download Center [44] which provides the official cartography of the Territorial Delimitations [54]. This information is provided in shp format (shapefile) in the Coordinate Reference System (in Spanish, SRC) ETRS 89, and of the Canary Islands in the coordinate reference system WGS 84.
  o The provinces area and the contour lines were combined in the QGIS software (step A.4.1.4) to define the corresponding climate zones. With this, we obtain a GIS map that represents the climate zones of Spain (Fig. 5).

##### 2.2.1.5 Step B1 – generation of energy performance model

This was composed of the following steps.

• **Step B1.1 - EPC data enhancement with the alphanumeric cadastre.** Each EPC was linked to the alphanumeric cadastre data through the cadastral references. This combination of data allowed us to obtain the use and built-up area of each certified building and BU.
• **Step B.1.2 – EPC outlier detection.** At this stage, we found that there are additional criteria to discard EPCs: some EPCs did not have a valid cadastral data, others were certifying a wrong use, and others were registered in the wrong Autonomous Community. All these EPCs were discarded. Further details about this process can be found in Appendix I, as well as the percentage of valid EPCs in each AC (Table A.9 and Table A.10) after this process.
• **Step B.1.3 – Classification of EPCs by main use.** The EPCs were classified by their main use in residential and non-residential. This was done for both, buildings and BUs. Since in the process of data management of the alphanumeric cadastre data, we already assigned a main use to buildings and BUs, it is possible to associate this information now to EPCs since they have been linked to the managed data from the alphanumeric cadastre.
• **Step B.1.4 – Group certificates by building.** A building can have a general EPC and/or one or several EPCs of the different BUs it is composed of (dwellings, commercial premises, offices, etc.). In our model we provide EPC information at two levels. On the one hand, we generated EPC information at the building level, given that this is the scale at which the final GIS model will work. For this purpose, an average value of energy consumption and CO_2_ emissions of the existing EPCs of each BU had to be calculated, when the building had no general EPC. On the other hand, we collected and kept in the model the information of the existing EPCs of the BUs, allowing us to have the original data of the energy performance of the dwellings, premises and buildings. To generate EPC information at the building level when no general EPC existed, we estimated the average non-renewable primary energy consumption and CO_2_ emissions as average values weighted by the built-up areas of the certified BUs, following (1), (2) in Appendix J.

As a result of step B1, we obtained a national EPC-based database by building and building unit with 2,966,479 EPCs, which correspond to 1,202,708 buildings, and certify a total of 3,811,061 BUs that sum up a total built-up area of 488,083,019 m^2^.

##### 2.2.1.6 Step B2 – generation of national enhanced building stock GIS model

This was composed of the following steps.

• **Step B.2.1 – Assignment of climate zone to each building.** To assign to each building its corresponding climate zone, the spatial join between the INSPIRE cadastre and the climate zone map was realised with QGIS.
• **Step B.2.2 – Combination of alphanumeric cadastre and INSPIRE information and climate zones.** The information from the alphanumeric cadastre and the result of step B.2.1 were merged to obtain an enhanced database with the information from both cadastres and the climate zone.
• **Step B.2.3. Assignment of municipality type.** According to the information on the population number in each municipality obtained from the Census (step A.4.2.2.), each building is assigned a municipality type depending on whether it is located in a rural or urban municipality

**Fig. 6:**
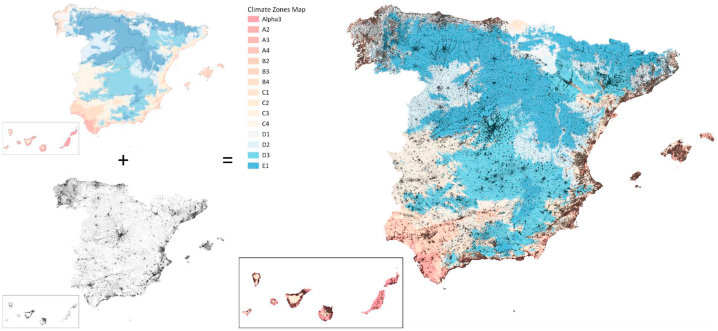
Georeferenced buildings in Spain with their Climate zone.

As a result of step B2, we obtained a national enhanced building stock GIS database with the general characteristics of the national building stock, as well as with data about the municipalities and climate zones where they are situated (Fig. 6). This model contains information of a total of 10,939,801 buildings, out of which 9,301,130 are buildings that require at least one EPC in one of their BUs because they have a certifiable use.

##### 2.2.1.7 Step B3 – generation of national-scale EPC-based UBEM

We obtained a national-scale EPC-based UBEM (result B.3. in Fig. 3) through the combination of the B1 and B2 models.

## 3 Results

### 3.1 Spain's EPC-based UBEM characteristics

**Fig. 7:**
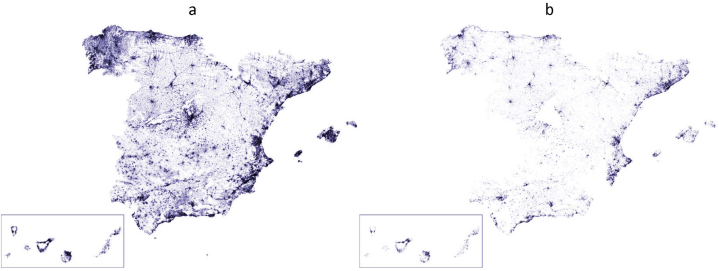
National-scale EPC-based UBEM showing, on the left panel, all the buildings in the model (7a), and, on the right panel, the buildings with at least one EPC (7b).

The generated model contains information about 10,939,801 buildings in Spain, out of which 1,202,708 have one or more EPCs (Fig. 7). The model allows us to map and analyse the buildings in the country by integrating multiple variables of different nature: geographical (AC, municipality, type of municipality), physical (area, number of floors, date of construction), use-related (main use of the building and use of each of its building units), energy-related (climate zone, energy class, energy consumption, CO_2_ emissions), among others. Therefore, this is a tool with a great potential to establish the renovation trajectory of the national building stock in Spain in response to the European minimum energy performance standards (MEPS). Additionally, it can also be used to establish a trajectory by regional and local administrations.

**Fig. 8:**
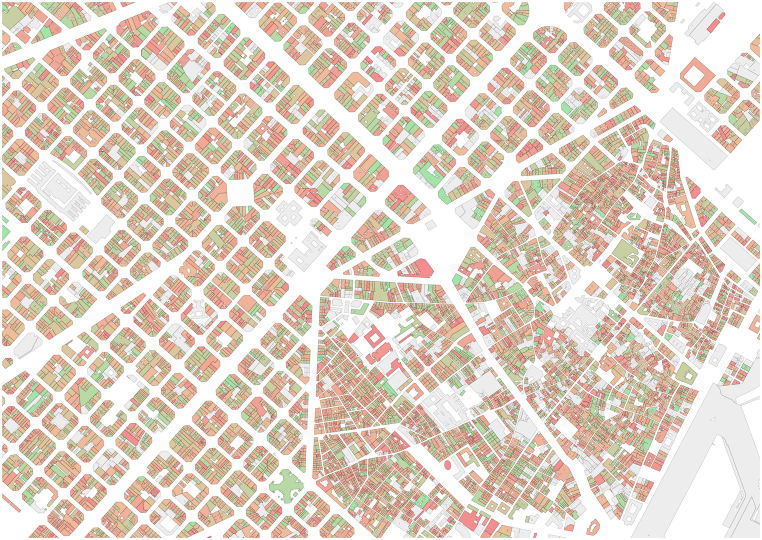
Buildings of the city centre of Barcelona ranked by NRPEC per square meter (green lower consumption to red higher consumption, grey: non-certified building). (For interpretation of the references to colour in this figure legend, the reader is referred to the Web version of this article.)

The degree of detail of the model reaches the building scale, so we can obtain information at this scale for all Spanish cities and villages. As example of this degree of detail, Fig. 8 shows the buildings of the city centre of Barcelona ranked by NRPEC per square meter.

### 3.2 Model validation

#### 3.2.1 Resources and time consumption to build the model

A key aspect in the generation of large-scale models is the consumption that they imply in terms of time and resources. As previously mentioned, a standard computer was used to generate the models described in this paper. The time consumption to automatically generate the models with this computer are shown in Table 3.

In order to obtain fast and light operation we paid special attention to the correct use of the formats. Due to the heterogeneity of the input data from the EPCs, the csv format was used to unify them. Once the data was homogenized and errors were filtered, the parquet format was used for the data management. This same parquet format was used for the manipulation of the alphanumeric cadastre and all the tabular data. For the generation of the models in GIS, the geoparquet format was used, due to its small size and reading speed. Table 4 shows a comparison of the calculation time based on the formats.

The reason why we did not automate step A.4.1 is because it does not require to be updated, unless the regulation that defines the climate zones changed.

#### 3.2.2 Robustness of the model

To check how correct the results from the model are, we selected four indicators and compared the results obtained for them with our model and the national data published by some official institutions.

##### 3.2.2.1 Indicators 1, 2 and 3 - number of buildings, dwellings and real estates

Table 5 shows the results for indicators number of buildings, dwellings and real estates.

The official data show a total of 11,130,090 built parcels. This is what in our model we have named buildings. Our model contains 10,934,241 buildings, and therefore it matches the official data in 98.2 %. The difference is mainly due to the fact that when we combined the alphanumeric cadastre, updated in real time, and the INSPIRE cadastre, updated every 6 months, we discarded the buildings that are not simultaneously in both DDBB. Additionally, some anomalous cases were also discarded because they share a cadastral reference as was explained in sections 3.1.2.3 and 3.1.3.3.

The cadastral statistics show 23,238,488 dwellings registered until 2019 and in our model there are 23,690,136 dwellings. The model has 1.9 % more dwellings than the cadastral statistics. This is due to the different time frames. The cadastral data is from 2019 and the model is from 2022.

The cadastre statistics provide 36,612,402 registered real estates, whereas the model contains 36,581,505. The difference is of 0.1 %. The difference is due to the combination of the INSPIRE cadastre and alphanumeric one, as well as due to the anomalous data.

The high percentages of coincidence regarding number of buildings (98.2 %), of dwellings (98.1 %) and of real estates (99.9 %) with official data show that the model is robust enough.

##### 3.2.2.2 Indicator 4 - percentage of buildings with different energy classes

The percentage of buildings with different energy classes was the fourth indicator. It is composed of two sub-indicators: non-renewable primary energy consumption (NRPEC) and CO_2_ emissions. Table 6 shows the values obtained for them with the national-scale EPC-based UBEM and the official data, as well as the absolute error. It must be noted that our model provides results for the period 2013–2022, whereas the official data provide results for 2013–2020.

The mean absolute error for all the energy classes for both sub-indicators is 0.7 %. This result supports the robustness of the national-scale EPC-based UBEM model. Even if our model does not contain the EPCs of certain ACs and has data updated to 2022, the results match the official data in 99.3 %.

### 3.3 Model usefulness

The model proposed here has been developed with the aim to support the definition of renovation trajectories by the European countries (in particular for the case of Spain), what will become compulsory when the 2023 EPBD recast gets finally approved [1]. This coming directive points out that MS are required to create a roadmap that includes nationally established targets, measurable progress indicators, and specific timelines. This roadmap will include the definition of a trajectory aimed at progressively reaching higher energy performance classes for all existing buildings. To explore the usefulness of this model, we verified that it is capable of generating some of the measurable progress indicators indicated as mandatory by the 2023 EPBD recast.

**Fig. 9:**
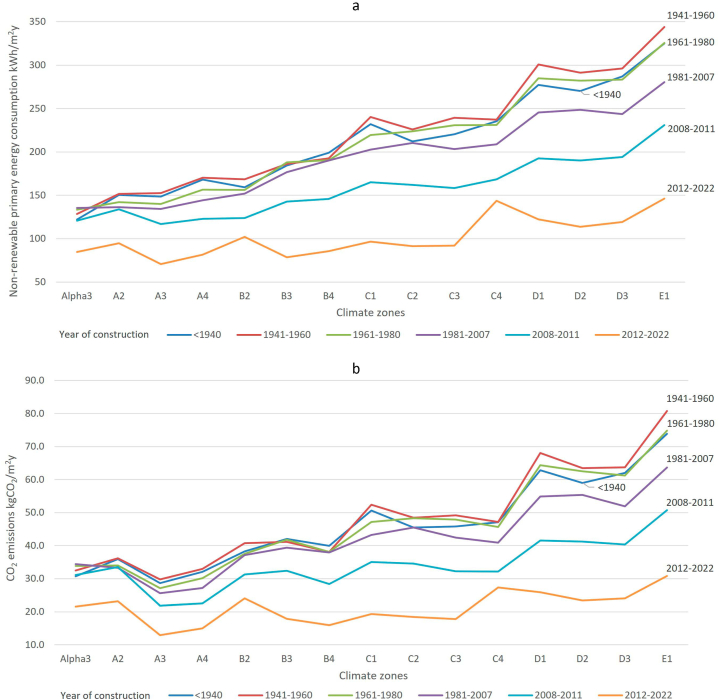
Non-renewable primary energy consumption (9a, upper panel) and CO_2_ emissions (9b, lower panel) of Spain's building stock per m2, climate zone and year of construction.

Two of the mandatory measurable indicators established in the 2023 EPBD recast is the **average non-renewable primary energy use in kWh/m**^**2**^**y** and the **annual greenhouse gas emission per building type**. The data depicted in Fig. 9 were obtained from our model. It shows the average NREPC and CO_2_ emissions per square meter and year, depending on the climate zone where it is located and the construction period in which it was built. For the period of construction of the buildings, the following clusters were considered: <1940, 1941–1960, 1961–1980, 1981–2007, 2008–2011, 2012–2022, as in Spain's Long-term renovation strategy. As can be seen in Fig. 9, the climate zone where the building is located has a strong influence on its energy consumption. The coldest areas of Spain, in climate zones with letters D and E, obtain 180 % higher levels of NREPC and CO_2_ emissions than the rest of areas of Spain. On the other hand, the gradual improvement of energy efficiency depending on the year of construction can be observed in all climate zones, especially in the 2008–2011 cluster, which corresponds to a period that begins with the approval of the Spanish Technical Construction Code, and the 2012–2022 one with the new version of the same regulation with a stronger commitment towards energy efficiency. The savings in NRPEC in the period 2012–2022 compared to the period before 1940 range from 31 % to 59 % depending on the climate zone and the savings in CO_2_ emissions between 30 % and 62 % in percentage, and between 9 and 43 in kgCO_2_/m^2^y. The coldest areas show the highest emissions saving potential. Therefore, the national renovation trajectory should take into consideration the importance that the coldest areas of Spain have in reducing emissions.

**Fig. 10:**
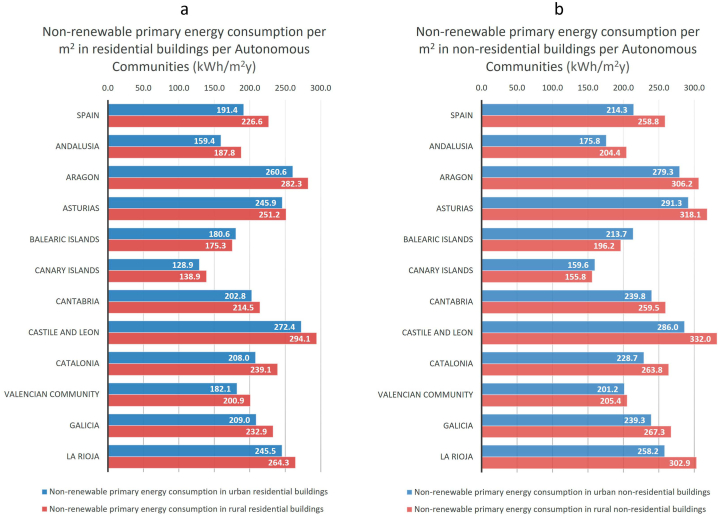
Non-renewable primary energy consumption of Spain's residential (10a, left panel) and non-residential (10b, right panel) building stock, per m^2^ and year, in urban and rural areas, and per Autonomous Community.

**Fig. 11:**
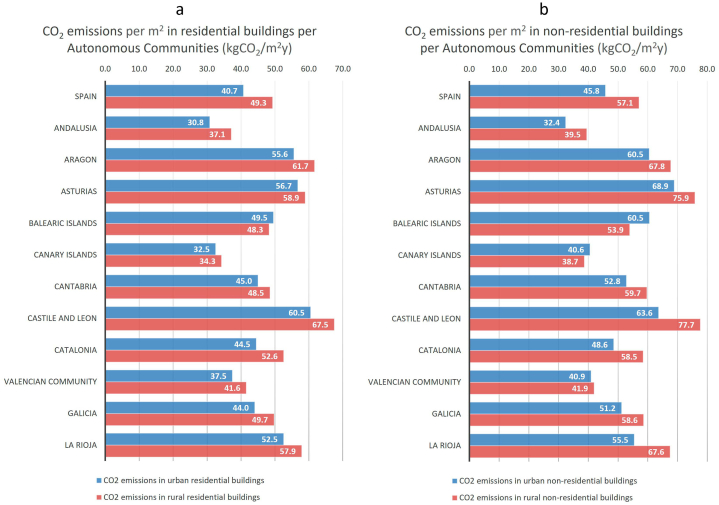
CO_2_ emissions of Spain's residential (11a, left panel) and non-residential (11b, right panel) building stock, per m^2^ and year, in urban and rural areas, and per Autonomous Community.

In addition to by climate zone, we can also extract the NRPEC and CO_2_ data geographically from our model. For example, in Fig. 10, Fig. 11 we have depicted the NRPEC and CO_2_ emissions, respectively, of Spain's residential and non-residential building stock per m^2^ and year, in urban and rural areas and per Autonomous Community. We can observe, in these Fig. 10, Fig. 11, that the distinction between ACs and between urban and rural areas allows us to identify specific problems. For example, the AC with the highest consumption is Castile and Leon, and the one with the lowest one is the Canary Islands. Furthermore, we observe that buildings located in rural areas have higher average consumption and emissions per m^2^ in Spain as a whole, as well as for the majority of the Autonomous Communities. For the whole of Spain residential buildings in urban areas consume in average 191.4 kWh/m2y, whereas in rural areas the consumption is 18.4 % higher (Fig. 10). With regard the average emissions of residential buildings, in urban areas it is of 40.7 kgCO_2_/m^2^y, and in rural areas it is 21.1 % higher (Fig. 11). For the whole of Spain non-residential buildings in urban areas consume in average 214.3 kWh/m^2^y (Fig. 10) and emit 45.8 kgCO_2_/m^2^y (Fig. 11), whereas in rural areas the consumption is 20.8 % higher (Fig. 10) and emissions 24.7 % (Fig. 11). These data must be considered when establishing priorities in the Spanish trajectory. The rural areas require a specific consideration in Spain.

**Fig. 12:**
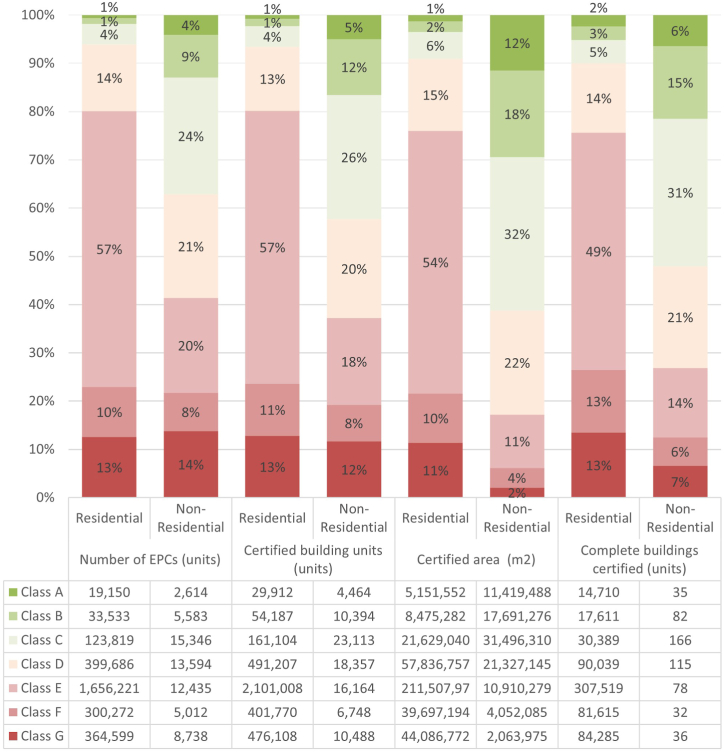
Number and percentage of EPCs, number and percentage of certified building units, certified area and percentage of certified area, and number and percentage of complete certified buildings, per energy class and use.

Others of the mandatory measurable indicators established in the 2023 EPBD recast are the **number of EPCs per energy class and number of EPCs per building type** and the **number of buildings and square meters per energy performance class**. In Fig. 12, we have depicted the number and percentage of EPCs, number and percentage of certified building units, certified area and percentage of certified area, and number and percentage of complete certified buildings, per energy class and use (residential and non-residential).

As can be seen in Fig. 12 in Spain's building stock, there is a very unequal distribution of energy classes, with the least efficient classes (E, F and G) representing the 80 % of the residential EPCs and the 42 % of the non-residential ones. The distribution of energy classes by area, building units and complete buildings is quite similar to that of EPC for the residential stock. However, the distribution is different for the non-residential stock. The certified area of non-residential buildings with the worst energy classes (letters E, F, and G) correspond to the 17 % of the certified area. This is mainly because the EPCs in the worst energy class most frequently belong to commercial premises on the ground floor of the buildings, with a small surface area.

It can be seen that there are more buildings with certificates for the entire building with better energy performance than building units, both for residential and non-residential stocks (Fig. 12). This is due to the fact that the new buildings are certified as a whole more often when they are sold, compared to the older buildings that are more frequently certified for individual real estates when they are rented.

All these data show the high degree of representation of the worst performing segments in Spain, especially for the residential stock, and the great challenge that Spain has to face to decarbonise its building stock. The renovation trajectory in Spain should focus on the most disadvantaged segments, and particularly on the residential use.

## 4 Discussion and conclusions

So far, national-scale Building Energy Models (UBEMs) have been generated based on archetypes and simulations. These types of models have been proven useful to estimate the energy consumption of national stocks [13,15]. However, their limitation is that they require the use of supercomputers mainly due to the energy simulation carried out on a large scale and they imply a simplification that underestimates the spread of energy performance.

In this paper we demonstrate that it is possible to generate a new type of UBEM, from EPCs, cadastral data and other open data, thus overcoming the limitations introduced by the use of archetypes and simulations, and with the advantage that the data can be updated automatically, in a short time, and with simple computer means. The reduced calculation time of our model is due to the fact that no energy simulations are made, but they are reused from the EPCs, taking advantage of the detailed simulations already realised by technicians. This new UBEM relies on GIS, whose connectivity and interoperability makes possible the reuse, combination and georeferencing of data from different sources, allowing us to cross-reference the existing information on our buildings on a case-by-case basis in order to carry out advanced energy studies of the building stock to define future trajectories for their energy renovation.

**Fig. 13:**
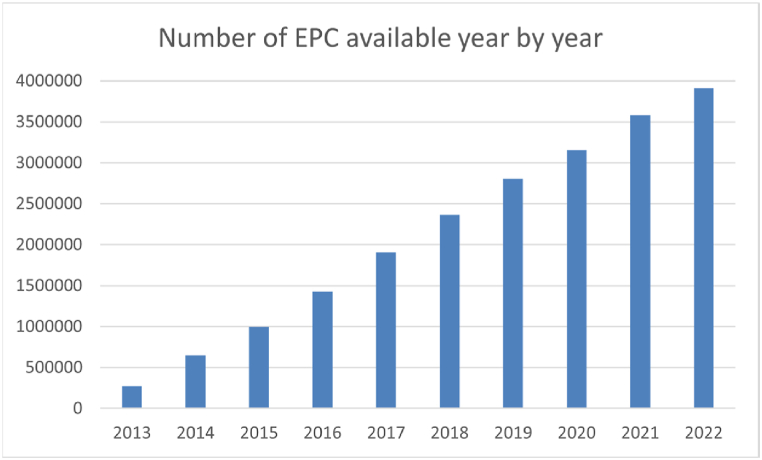
Number of EPCs per year in the open databases in Spain.

Even if EPCs require significant outlier detection, as has been observed in the case study of Spain, they have the advantage of providing access to a large number of energy simulations carried out in detail and building-by-building by technicians. Additionally, it must be noticed that the potential of EPCs is growing due to the increase in the number of certificates openly available (Fig. 13). Up to January 1, 2023, there are 3,920,354 EPCs with open data in Spain.

### 4.1 Limitations of the model

The model generated here has some limitations, deriving most of which from some gaps in the data from the sources used. Each country will have its own limitations depending on the source data. The limitations for the case of Spain are listed below.

• The INSPIRE cadastre and the alphanumeric cadastre do not include building ownership data for reasons of personal data protection. As a consequence, it is not possible to incorporate the property type information (private or public) for each building. This information would be useful to be able to carry out advanced energy analyses of public and private buildings separately, and to define different renovation trajectories for them. In this sense, it would be convenient if the cadastre could provide the information regarding type of property for research purposes. However, it must be noticed that for the case of Spain, given that publicly owned housing units in Spain only account for 1.5 % of the total [56] the model is considered valid for the diagnosis of private housing and the design of its linear trajectory.
• At the moment, the number of certified buildings is low (12.93 %) compared to the existing total. However, it can be seen in Fig. 13 that the number of EPCs significantly increases each year. This, together with the fact that the European directives continue to rely on the EPC as one of the main tools to help improve the energy performance of buildings, make us think that this type of model will be fed by an increasing amount of data in the future.
• Some ACs in Spain do not openly publish the results of the EPCs yet. However, we know that the 2023 EPBD recast specifies that the EPC databases must be publicly available, machine-readable, accessible via a digital interface and interoperable with administrative databases such as cadastre or digital building logbook. Therefore, it can be expected that in the more or less near future all the ACs will make them public.
• The EPCs are based on energy simulation and do not accurately estimate the real energy consumption of the buildings. This is the case, among other issues, because factors, such as user behaviour, influence actual energy consumption [57]. However, even if this is true, energy certificates represent a homogeneous way of evaluating the building stock and assign, for example, consumptions to dwellings that, due to energy poverty, would not actually have them. For this reason, we consider that the use of EPCs is appropriate for the design of renovation trajectories, since they allow us to evaluate the existing buildings with homogeneous energy efficiency criteria, allowing us to detect the worst-performing cases, which are not hidden by issues related to user behaviour.
• The lack of homogenization of the EPC data published by the different ACs of Spain is a serious barrier to be able to make a broader use of the data. For this reason, we recommend standardizing the data that is published and its format.

### 4.2 Conclusions and future research

In this paper we have defined a methodology to build a national-scale EPC-based UBEM, an automated model which uses open data from sources established at a European scale (EPC, INSPIRE cadastre and DTM) and other national open data (alphanumeric cadastre, national laws, and national statistics), to build a single GIS database useful for the energy diagnosis of national building stocks and to establish national renovation trajectories in Europe.

The model has been proven to be automatically built and updated. The automated processes to build the model took in total less than 6 h for the country of Spain with a standard computer. The model allows to map and analyse the buildings in the country by integrating multiple variables of different nature, such as geographical, physical, use-related, and energy-related.

The robustness of the model has been tested for four indicators and it has been verified that it provides results that match official data in more than 98 %.

To study the usefulness of the model, some of the indicators that the future 2023 EPBD recast will establish as mandatory have been developed. From the development of these indicators, through the use of our model, it has been seen that the areas in Spain with cold winters (corresponding to climate zones D and E) have a high importance in the decarbonisation of the building stock in this country since they obtain levels of NREPC and CO_2_ emissions 180 % higher than other climate zones. It has also been observed that the buildings in rural areas have higher consumptions (18.4 % for residential buildings and 20.8 % for non-residential) and emissions (21.1 % for residential buildings and 24.7 % for non-residential). Finally, it was observed that the residential park in Spain requires a significant energy improvement, since 75 % of the energy-certified residential surface is among the worst energy classes corresponding to the letters E, F, and G. These data, which were not available in Spain until the generation of this model, and many others that can be generated, support the usefulness of the developed model.

In the future, we will further test the usefulness of the model to diagnose the national building stock and to design linear renovation trajectories. Jiménez Solana [58] points out that with this type of models, the data could even be reused to estimate the energy efficiency of the entire national building stock, in a similar way to how massive cadastral valuation procedures are carried out, at a free cost for citizens. We will study how to incorporate an estimation of the energy efficiency of the buildings that still do not have an EPC. Additionally, it would be interesting to study in the future the interoperability of the information of this model with other similar models of different European countries.

## Additional information

No additional information is available for this paper.

## Data availability statement

The code created for generating the national-scale EPC-based UBEM is available together with this paper on GitHub at https://github.com/CarlosBeltranVelamazan/NBEM.

## CRediT authorship contribution statement

**Carlos Beltrán-Velamazán:** Writing – original draft, Visualization, Validation, Software, Methodology, Investigation, Formal analysis, Data curation, Conceptualization. **Marta Monzón-Chavarrías:** Writing – review & editing, Writing – original draft, Supervision, Resources, Methodology, Conceptualization. **Belinda López-Mesa:** Writing – review & editing, Supervision, Resources, Project administration, Methodology, Funding acquisition, Conceptualization.

## Declaration of competing interest

The authors declare the following financial interests/personal relationships which may be considered as potential competing interests: Belinda Lopez-Mesa reports financial support was provided by Spain 10.13039/501100004837Ministry of Science and Innovation. Belinda Lopez-Mesa reports financial support was provided by Government of Aragón. If there are other authors, they declare that they have no known competing financial interests or personal relationships that could have appeared to influence the work reported in this paper.

## Figures and Tables

**Table A.1:** Autonomous communities in Spain that share the EPCs in open databases

Autonomous Communities	Open data available	References
Andalusia	YES	[61]
Aragon	YES	[62]
Asturias	YES	[63]
Balearic Islands	YES	[64]
Basque Country	NO	–
Canary Islands	YES	[65]
Cantabria	YES	[66]
Castile and León	YES	[67]
Castilla-La Mancha	YES	[68]
Catalonia	YES	[69]
Comunitat Valenciana	YES	[70]
Extremadura	NO	–
Galicia	YES	[71]
Rioja	YES	[72]
Madrid	NO	–
Murcia	NO	–
Navarra	YES	[73]
Ceuta	NO	–
Melilla	NO	–

**Table A.2:** Data obtained from each of the sources of information (CNIG: National Centre for Geographic Information; DB HE: Basic Document on Energy Saving; DTM: Digital Terrain Model; EPC: Energy Performance Certificates; INSPIRE: Infrastructure for Spatial Information in Europe; NRPEC: Non-renewable primary energy consumption).

Data obtained from EPC	Data obtained from alphanumeric cadastre	Data obtained from INSPIRE cadastre	Data obtained from additional sources of information (source and data)
● Cadastral reference ● Certificate registration date ● NRPEC ● Energy class of NRPEC ● CO_2_ emissions ● Energy class of CO_2_ emissions ● AC where the EPC was recorded	● Cadastral reference ● Use of the building and building unit ● Certifiable area per building and building unit ● Number of properties comprising a building requiring an EPC ● Municipality, autonomous community and province ● Number of floors	● Cadastral reference ● Georeferenced buildings	● Census: number of inhabitants per municipality ● MDT 200: altitude of each plot and contour lines ● DB HE: climate zone by province ● CNIG: map of provinces

**Table A.3:** Generated models and data (AC: Autonomous Community; EPC: Energy Performance Certificate; NRPEC: Non-renewable Primary Energy Consumption)

Model	B1. Energy performance model	B2. National enhanced building stock GIS model	B3. National-scale EPC-based UBEM
Information scale	● Building (tabular format) ● Building unit (tabular format)	● Building (GIS format)	● Building (GIS format) ● Building unit (tabular format)
Data the model provides	● Cadastral reference ● Energy performance (NRPEC and CO_2_) ● Energy class ● Certified Use ● Certified surface ● Number of certified building unit in the same building ● Number of EPC in a building	●Cadastral reference ●Georeferenced buildings ●Main use of the building ●Number of building units that make up each building ●Number of building units by use ●Built-up area of each use ●Year of construction ●Number of floors ●AC, province, municipality ●Climate zone	● Cadastral reference ● Georeferenced buildings ● Main use of the building ●Number of building units that make up each building ●Number of building units by use ●Built-up area of each use ●Year of construction ●Number of floors ●AC, province, municipality ●Climatic zone ● Energy performance (NRPEC and CO_2_) ● Energy class ● Certified Use ● Certified surface ● Number of certified building unit in the same building ● Number of EPC in a building

**Table A.4:** EPC information in open data provided by each AC. Blank: it does not contain this information; Y: it contains this information; P: it partially contains this information.

Region	Data to locate the EPC						Type of building			Energy and emissions data					Other relevant data		Total out of 16
	Cadastral Reference	Postal Code	Coordinates X, Y	Municipality Code	Municipality	Climate Zone	Type of building	Built date	Area	NRPEC	Letter label according to NRPEC	CO_2_ emissions	Letter label according to CO_2_ emissions	Type of heating, refrigeration, DHW	Regulations, software, energy type	Certificate registration date	
Andalusia	Y	Y			Y	Y	Y	Y	Y	Y	Y	Y	Y	Y	Y	Y	14
Aragon	Y		Y		Y		Y	Y	Y	Y	Y	Y	Y			Y	10
Asturias	Y	Y			Y	Y	Y	Y	Y	Y	Y	Y	Y	Y	Y	Y	14
Balearic Islands	Y	Y			Y		Y	Y	Y	Y		Y	P		P	Y	11
Canary Islands	Y				Y	Y				Y	Y	Y	Y	Y		Y	9
Cantabria	Y	Y			Y	Y				Y	Y	Y	Y	Y		Y	10
Castile and Leon	Y	Y	Y		Y		Y			Y	Y	Y	Y		P	Y	11
Castilla La Mancha	Y	Y	Y		Y		Y			Y	Y	Y	Y		P	Y	6
Catalonia	Y	Y		Y	Y	Y	Y		Y	Y	Y	Y	Y		Y	Y	13
Valencian Community	Y	Y			Y		Y	Y		Y	Y	Y	Y			P	10
Estremadura																	–
Galicia	Y	Y			Y		Y			Y	Y	Y	Y		P	Y	9
Madrid																	–
Murcia																	–
Navarre		Y			Y					Y	Y	Y	Y		P		7
Basque Country																	–
Rioja	Y		Y	Y	Y	Y	Y	Y	Y	Y	Y	Y	Y		P	P	14
Ceuta																	–
Melilla																	–
Total out of 12	12	10	3	2	12	6	10	6	6	12	12	12	12	4	8	10	

**Table A.5:** File formats and encodings in which the information is published in each AC.

File format	Autonomous Communities (AC)
CSV, encoding = "UTF-8"	Aragon, Castila and Leon, Valencian Community, Navarra
CSV, encoding = "UTF-8 with BOM"	Baleares, Catalonia
CSV, encoding = "ANSI"	Canarias, Cantabria, Galicia, Rioja
XLSX, encoding = "UTF-8"	Aragon, Asturias, Cantabria,
XML, encoding = "UTF-8"	Andalusia, Aragon, Rioja
XML, encoding = "ANSI"	Galicia
XLS, encoding = "UTF-8"	Castilla and Leon, Valencian Community
XLS, encoding = "ANSI"	Castilla La Mancha, Rioja
JSON encoding = "UTF-8"	Aragon, Castilla and Leon, Rioja
Shp or geojson	Catalonia

**Table A.6:** Criteria used to reject EPC with incorrect information.

Criteria to filter the data	Threshold
Non-renewable primary energy consumption	≤629,7 kWh/m^2^·y
Non-renewable primary energy consumption class	A letter from A to G
CO_2_ emissions	≤333,1 kgCO_2_/m^2^·y
CO_2_ emissions class	A letter from A to G
Certificate registration date	Between January 01, 2013 and January 01, 2023 (EPCs in force)
Eliminate duplicated EPCs	Only the most recent is valid

**Table A.7:** Information useful for this study obtained from the Alphanumeric Spanish Cadastral data in categories parcel, construction unit, real estate, and construction.

Parcel	Construction unit	Real Estate	Construction
● Cadastral parcel reference ● Province and municipality data ● Postal code ● Plot area ● Built-up area ● Built-up area below ground level ● Built-up area above ground level ● Roof area ● X and Y coordinates of plot centroid	● Cadastral parcel reference ● Province and municipality data ● Year of construction ● Level of accuracy of year of construction ● Area of construction unit footprint ● Façade length	● Cadastral real estate reference ● Province and municipality data ● Real estate year of constrution ● Number of floors ● Use of the real estate	● Cadastral real estate reference ● Province and municipality data ● Area of the construction ● Year of construction ● Number of floors ● Use of the construction (destination code) ● Renovation indicator ● Year of renovation ● Effective longevity in cadastre ● Porches and terraces area ● Other floors area

**Table A.8:** Categories from the cadastre used to enrich the data scales building and building unit in the national-scale EPC-based UBEM.

Data scale	Alphanumeric cadastral categories
Buildings	Parcel, Construction unit, Real estates, Constructions
Building units	Real estates, Constructions

**Table A.9:** Criteria to reject EPCs due to incorrect information

Criteria to filter the data	Threshold
The EPCs must have an existing cadastral reference	Non-specified, non-existing in the alphanumeric cadastre, or cadastral reference with errors
The EPCs must have a certifiable use	Non-certifiable uses: storage-parking use, urbanization works, agriculture, industrial, industrial-agricultural, and agricultural storage
The EPC must be registered in the same AC as the building	The building is situated in a different AC to that where it was registered

**Table A.10:** Percentage of valid EPCs in each AC after applying outlier detection criteria.

AC	Number of registered EPCs	Number of EPCs after outlier detection	Percentage of valid EPCs
Andalusia	548,359	490,219	89.40 %
Aragon	161,830	145,688	90.03 %
Asturias	59,807	28,588	47.80 %
Balearic Islands	158,678	69,777	43.97 %
Canary Islands	189,450	105,726	55.81 %
Cantabria	83,544	56,305	67.40 %
Castile and Leon	202,643	169,181	83.49 %
Castilla-La Mancha	146,000	117,760	80.66 %
Catalonia	1,371,455	1,041,595	75.95 %
Valencian Community	711,988	583,348	81.93 %
Galicia	183,939	124,025	67.43 %
Rioja	42,604	34,267	80.43 %
Navarra	60,057	0	0.00 %
TOTAL	3,920,354	2,966,479	75.67 %

## Appendix D Further details of step A.1.1: EPC bulk download

Table A.4 shows the EPC information in open format provided by the ACs divided in four categories: data to geolocate the building, information about the building, energy data of the building and other relevant data from the certificate. As can be seen, the heterogeneity of the EPC open data in Spain is high. The region of Balearic Islands publishes an energy efficiency letter but does not specify which indicator it belongs to. In this study, we presumed the letter refers to indicator CO_2_ emissions because it has traditionally been considered the main one.

## Appendix E Further details of step A.1.2: Data pre-processing: homogenization

Table A.5 shows the format of the files provided by the different ACs. Some ACs provide the information in more than one format.

## Appendix F Further details of step A.1.3: Outlier detection in EPCs

As suggested by Ref. [59], the criterion to select the NRPEC and CO_2_ emissions thresholds is using as a reference value 1.5 times the threshold of the most unfavourable letter G in Spain. The threshold of CO_2_ emissions is obtained multiplying the energy threshold by the most unfavourable energy carrier [60].

For this study, the EPCs as of January 1, 2023 were considered, and the EPCs which are no longer in force were discarded. This happens for EPCs of over ten years and certificates that have been updated with a more recent one.

## Appendix I Further details of step B.1.2: Outlier detection

The criteria used to reject EPCs due to incorrect information are shown in Table A.9. Next, we provide an explanation for the specified criteria.

In order to link the alphanumeric cadastre data to the EPCs, the latter must have a valid cadastral reference. The EPCs that contained errors in the reference were discarded. The EPCs of the AC of Navarra were all left out of this study, as they are not part of the Spanish cadastre.

The alphanumeric cadastre differentiates between sixteen uses of real estates (or BUs in our model). Six of these uses out of them do not require an EPC, in accordance to RD 390/2021, of June 1, which approves the basic procedure for the certification of the energy efficiency of buildings in Spain. Those six uses are shown in Table A.9. We found that some EPCs were certifying this type of uses, and since this is an error, they were also discarded. The EPCs that were included in this study correspond to the following ten certifiable uses: residential, offices, commercial, sports, shows, leisure and hostelry, health and charity, cultural, religious and singular buildings.

Some certificates were found to be registered in the wrong Autonomous Community. These EPCs were also discarded since according to regulation EPCs must be registered in the AC where the building is located.

As a result of the outlier detection process applying the criteria shown in Table A.6 and Table A.9, 2,966,479 EPCs were found to be valid, representing the 75.67 % of the EPCs. These EPCs, which may be applied to a building or BU, correspond to a total of 1,202,708 buildings with at least an EPC, representing the 12.93 % of existing buildings in Spain with a certifiable use. Table A.10 shows the number and percentage of valid certificates per AC.

## Appendix J Formulae to estimate the average non-renewable primary energy consumption and CO_2_ emissions of buildings with no general EPC and one or several EPCs of its BUs

(1) $$C_{Building}=\frac{\sum_{n=1}^{N}\left(C_{BU}*S_{BU}\right)}{S_{nBU}}$$

(2) $$E_{Building}=\frac{\sum_{n=1}^{N}\left(E_{BU}*S_{BU}\right)}{S_{nBU}}$$

Where:

$$C_{Building}=Average\;NRPEC\;of\;the\;building$$

$$C_{BU}=NRPEC\;of\;each\;certified\;BU$$

$$E_{Building}=Average\;{CO}_{2}\;emissions\;of\;the\;building$$

$$E_{BU}={CO}_{2}\;emissions\;of\;each\;certified\;BU$$

N=totalnumberofEPCs

$$S_{BU}=built-up\;area\;of\;each\;certified\;BU$$

$$S_{nBU}=Total\;built-up\;area\;of\;all\;certified\;BUs$$
